# Temperature-Dependent Photoluminescence of Manganese Halide with Tetrahedron Structure in Anti-Perovskites

**DOI:** 10.3390/nano11123310

**Published:** 2021-12-06

**Authors:** Yijie Xia, Shuaishuai Du, Pengju Huang, Luchao Wu, Siyu Yan, Weizhi Wang, Gaoyu Zhong

**Affiliations:** 1 School of Mechanical Engineering, University of Shanghai for Science and Technology, Shanghai 200093, China; xiayj@usst.edu.cn (Y.X.); 192341371@st.usst.edu.cn (S.D.); 202291491@st.usst.edu.cn (P.H.); 2 Department of Materials Science, Fudan University, Shanghai 200433, China; 21110300059@m.fudan.edu.cn; 3 Collaborative Innovation Center of Polymers and Polymer Composite Materials, State Key Laboratory of Molecular Engineering of Polymers, Department of Macromolecular Science, Fudan University, Shanghai 200433, China; 18210440024@fudan.edu.cn (S.Y.); weizhiwang@fudan.edu.cn (W.W.)

**Keywords:** [MnBr_4_]BrCs_3_, temperature-dependent, photoluminescence, excitons

## Abstract

The temperature-dependent photoluminescence (PL) properties of an anti-perovskite [MnBr_4_]BrCs_3_ sample in the temperature range of 78–500 K are studied in the present work. This material exhibits unique performance which is different from a typical perovskite. Experiments showed that from room temperature to 78 K, the luminous intensity increased as the temperature decreased. From room temperature to 500 K, the photoluminescence intensity gradually decreased with increasing temperature. Experiments with varying temperatures repeatedly showed that the emission wavelength was very stable. Based on the above-mentioned phenomenon of the changing photoluminescence under different temperatures, the mechanism is deduced from the temperature-dependent characteristics of excitons, and the experimental results are explained on the basis of the types of excitons with different energy levels and different recombination rates involved in the steady-state PL process. The results show that in the measured temperature range of 78–500 K, the steady-state PL of [MnBr_4_]BrCs_3_ had three excitons with different energy levels and recombination rates participating. The involved excitons with the highest energy level not only had a high radiative recombination rate, but a high non-radiative recombination rate as well. The excitons at the second-highest energy level had a similar radiative recombination rate to the lowest energy level excitons and a had high non-radiative recombination rate. These excitons made the photoluminescence gradually decrease with increasing temperature. This may be the reason for this material’s high photoluminescence efficiency and low electroluminescence efficiency.

## 1. Introduction

Metal halide perovskites have recently attracted widespread attention due to their excellent optical and electronic properties, such as high carrier mobility [1,2], saturated emission color [3,4], and easy color adjustability [5,6]. In particular, the external quantum efficiency (EQE) of organic–inorganic hybrid lead bromide perovskite green light-emitting perovskite LEDs (PeLEDs) has made a breakthrough, recently breaking through about 20% [7,8]. The flexible tunability of the organic–inorganic hybrid perovskite structure gives it a variety of crystal structures and superior performance, including high charge injection and transport capabilities, high photoluminescence (PL) quantum yield, narrow full width at half maximum (FWHM), as well as defect tolerance behavior [9]. Unfortunately, mixed perovskites containing a small number of organic cations such as methylammonium (MA) or formamidine (FA) are very sensitive to water, which leads to the rapid decline of LED performance [10,11], thus limiting their prospects in practical applications. Similar to inorganic hybrids, the perovskite CsPbX_3_ (X = Cl, Br, or I) based on inorganic cesium cations shows better thermal and chemical stability [12,13]; however, lead’s inherent toxicity and the instability of lead-based perovskites severely limit their practical applications. Therefore, there is a great demand for the development of lead-free perovskite materials. Researchers are working to find lead-free perovskites to replace toxic lead with divalent metal ions such as Sn^2+^ and Ge^2+^. 

Unfortunately, due to the oxidation of Sn^2+^ and Ge^2+^, the corresponding materials have been shown to be unstable [14,15]. Because of this, many researchers have turned their attention to manganese(II)-based perovskites, which have additional unique properties (such as multi-iron, phase change memory, nonlinear optical molecules, and tunable luminescence), and the coexistence of multiple expressions has been observed in previous works [16,17,18].

Compared with typical perovskite, anti-perovskite has the opposite structure. The general formula is [MX_4_]XA_3_ (A (I) = alkali metal; M (II) = transition metal; X = Cl, Br, I). As the luminescence center, a [MX_4_] tetrahedron is separated by a three-dimensional (3D) XA_6_ octahedral anti-perovskite framework. This unique structure can effectively reduce the interaction degree of luminescence centers and increase the spatial constraint effect so that these materials have high PLQY and luminescence color stability. Yan et al. [19] prepared perovskite thin films by using the double-source thermal evaporation method, and prepared the first all-inorganic cesium manganese halide anti-perovskite light-emitting diode, with a maximum external quantum efficiency of 12.5%, maximum luminous brightness of 3990 cd/m^2^, and half-life of 756 min at 5.0 V.

However, some basic issues still need to be resolved. For example, the temperature dependence of pump excitons is still unclear. In addition, temperature-dependent PL spectra can also provide insight into the photophysical properties of materials, even those that contain complex structures, such as core/shell heterostructures. Recently, Lee et al. [20] used temperature-dependent steady-state and time-resolved PL to study the thermal quenching behavior and carrier interaction of CsPbBr_3_ quantum dots of different sizes. 

For [MnBr_4_]BrCs_3_, we tried many methods to prepare electroluminescent devices, but electroluminescence was still difficult to achieve. We think this may be related to the exciton characteristics involved in luminescence. Therefore, in the present work we study the exciton characteristics involved in luminescence by changing the temperature of photoluminescence and attempt to explain the internal mechanism of its difficulty in realizing electroluminescence.

In this research, the steady-state photoluminescence spectra of a [MnBr_4_]BrCs_3_ thin film measured at temperatures ranging from 78 to 500 K were studied. The variation of PL intensity with temperature was derived, which was based on the Boltzmann distribution and dynamic equilibrium among the types of excitons with different energy levels and different recombination rates involved in the steady-state PL process. The derived expression was in good agreement with the experimental data. At the same time, the fitting parameters provided a great deal of physical information for the experimental results. The results of the photoluminescence experiments showed that there were three main types of excitons involved in the luminescence process—that is, the light-excited excitons still tended to recombine whether light was emitting or not. It seemed difficult to form carriers by the disconnection of electrons and holes in the exciton, meaning that it was difficult to realize electroluminescence, which originated from the radiative recombination of excitons formed from the attracted free electron and hole.

## 2. Materials and Methods

### 2.1. Material Preparation

Cesium bromide (CsBr, 99.5%, metal based) was purchased from Aladdin reagent Co., Ltd. (Shanghai, China). Anhydrous manganese bromide (MnBr_2_, AR) was purchased from Changsha Jingkang New Material Technology Co., Ltd. (Changsha, China). Acetone (AR) and absolute ethanol (AR) were purchased from Sinopharm Chemical Reagent Co., Ltd. (Shanghai, China). All materials were used without further purification. [MnBr_4_]BrCs_3_ was synthesized by the vacuum solid-phase reaction method, which was very important, because it can prevent the hygroscopic effect of [MnBr_4_]BrCs_3_ halide, but also prevent H_2_O coordination during the crystallization process. CsBr and MnBr_2_ were weighed at a stoichiometric molar ratio of 3:1 and sealed in a vacuum quartz tube. In a tube furnace, it was heated to 720 °C with a temperature gradient of 5 °C/min and maintained at 720 ± 50 °C for 120 min (to ensure that the raw materials were in a molten state), and then cooled to room temperature at a rate of −0.2 °C/min.

### 2.2. The Preparation of [MnBr_4_]BrCs_3_ Films

A clean polished silicon wafer was used as the substrate. The silicon wafers were continuously ultrasonically rinsed in acetone, absolute ethanol, and deionized water for 10 min. After drying with nitrogen, a film of [MnBr_4_]BrCs_3_ was deposited on the substrate through a vacuum evaporation system. During the deposition process, the pressure of the evaporation chamber was better than 2 × 10^−4^ Pa, the sample thickness was 500 nm, and the evaporation rate was ~1 nm/s. After the film deposition was complete, the sample was transferred to another chamber.

### 2.3. Characterization of [MnBr_4_]BrCs_3_

Single-crystal X-ray diffraction data of [MnBr_4_]BrCs_3_ were collected using a Bruker D8 Venture single-crystal X-ray diffractometer (Beijing, China). The diffraction pattern angular range was 5–50° (2θ) with a step size of 0.02° at room temperature. Calculated powder patterns were simulated by Mercury software using the crystallographic information files (CIFs) from single-crystal X-ray experiments. Thermogravimetric analysis (TGA) of the single crystal was evaluated using a synchronous thermal analysis-mass spectrometry system (SDT Q600-GSD 301 T2, TA-Pfeiffer, Beijing, China) at a heating rate of 10 °C/min in an N_2_ atmosphere.

### 2.4. Temperature Dependence Experiment

Temperature-dependent PL measurement was performed using a vacuum liquid nitrogen cryostat with temperature control. The excitation light source was a 325 nm helium-cadmium laser beam (Melles Griot 56 He-Cd series, Suzhou, China) and the Ocean Optics USB4000 fiber optic spectrometer (Ocean Insight, Shanghai, China) was used for spectrum collection and measurement.

## 3. Results and Discussion

**Figure 1:**
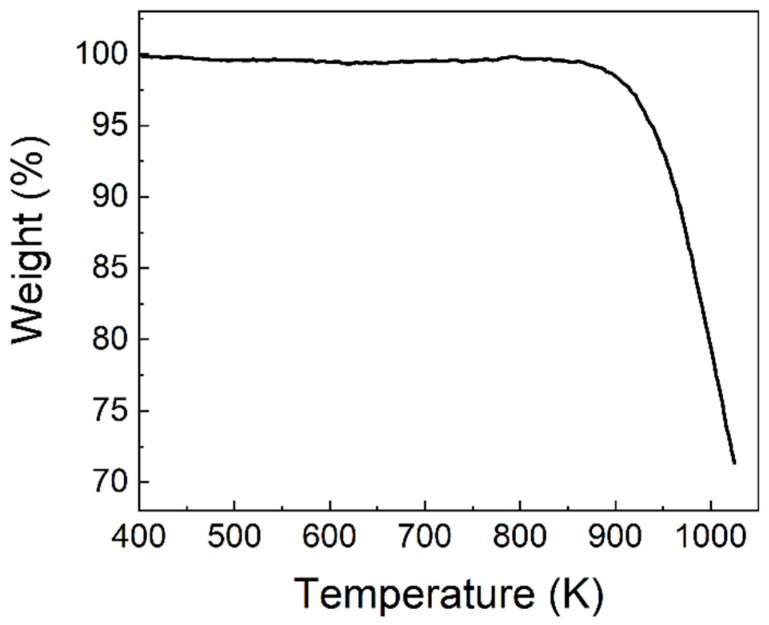
TGA of anti-perovskite [MnBr_4_]BrCs_3_ film.

**Figure 2:**
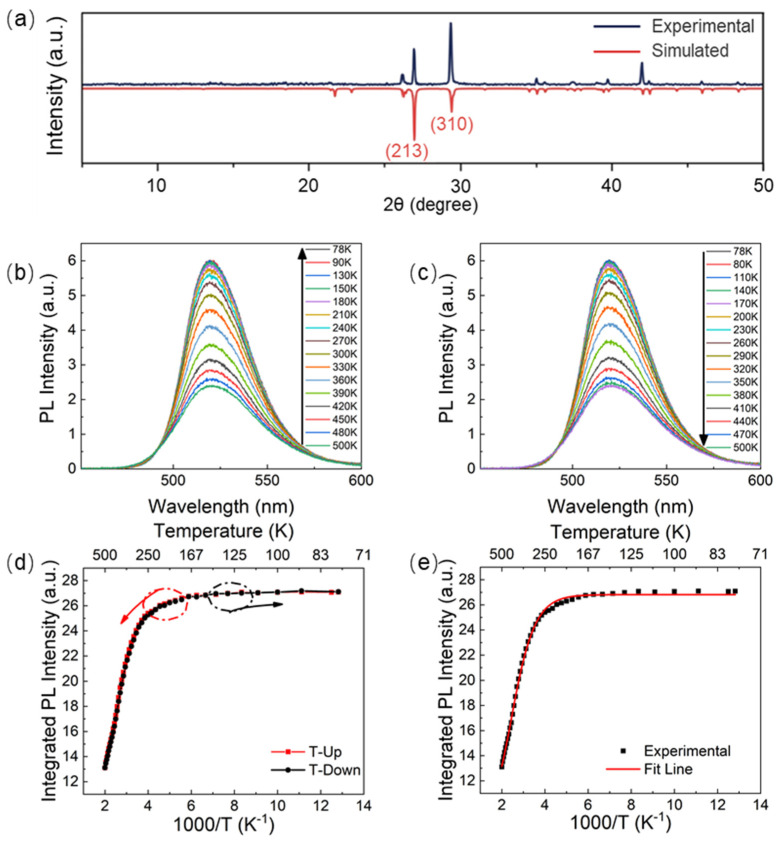
(**a**) Experimental XRD pattern (deep blue curve) and calculated XRD pattern (red curve) of [MnBr_4_]BrCs_3_ film. The experimental XRD pattern was measured by PXRD and the calculated XRD pattern was simulated by Mercury software using the crystallographic information files (CIFs) from single-crystal X-ray experiments. (**b**) The PL spectra with decreasing temperatures ranging from 500 to 78 K. (**c**) The PL spectra with increasing temperatures ranging from 78 to 500 K. (**d**) The integrated PL intensity as a function of 1000/T for [MnBr_4_]BrCs_3_ film. The solid red line shows the heating process. The cooling process is shown as the solid black line. (**e**) The integrated PL intensity vs. temperature reciprocal of the [MnBr_4_]BrCs_3_ thin films. Solid squares represent the experimental results and the solid curve represents the fit.

The material [MnBr_4_]BrCs_3_ had good thermal stability, which can be seen from Figure 1, because the loss of single crystal at 938 K in the TGA experiment was only 5%. As shown in Figure 2a, (213) and (310) characteristic diffraction peaks can clearly be seen in the experimental XRD pattern, which was almost in accord with the calculated one, confirming the reliability of the result and that the [MnBr_4_]BrCs_3_ films were single crystalline. Under the excitation wavelength of 325 nm, the changes of photoluminescence spectrum of [MnBr_4_]BrCs_3_ film measured at 78–500 K were studied, as shown in Figure 2b,c. As shown in Figure 2b, the PL intensity of [MnBr_4_]BrCs_3_ decreased with increasing temperatures. From room temperature to 78 K, due to the exciton–photon coupling in [MnBr_4_]BrCs_3_, excitons that changed by temperature participated in the light emission, resulting in different spectra, narrowing the emission full width at half maximum, reducing the thermal vibration at low temperatures, and gradually increasing the emission intensity. From room temperature to 500 K, as the temperature increased, the PL intensity gradually decreased.

These photoluminescence spectra with varying temperatures show the trend of the luminescence intensity changing with temperature. More tests would be needed in order to understand the mechanism involved in the multiple-peak photoluminescence. For example, the measurement of excitation spectra is necessary to know the involved excitons with different energy states and different radiative recombination rates. In our previous work [19], we measured the excitation spectra of a series of [MX_4_] tetrahedral perovskites with different halogen elements. The wavelength range of the pumping light was 250~480 nm. We found that the wavelength of the light radiated by this series of materials was always in the range of 450~620 nm when excited, indicating that these materials emit many lights with several fixed wavelengths and different intensities. Similar studies can be found in [21]. The excited energy states and photoluminescence efficiencies of many crystals were obtained by studying the photoluminescence spectra pumped by many lights.

**Figure 3:**
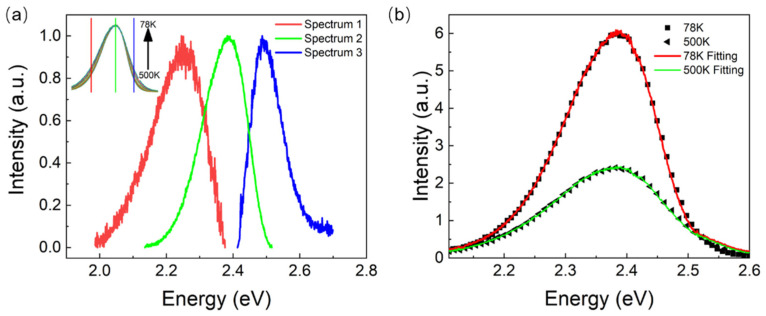
(**a**) Three resolved spectra extracted from the temperature-dependent PL spectra. The inset shows the normalized temperature-dependent spectra, and the solid lines with three colors indicate the three resolved normalized spectra. (**b**) The measured and fitted PL spectra at 78 K and 500 K, which is the linear combination of the three resolved spectra. The solid line shows the fitted curve.

It can be seen from Figure 2b,c that the temperature-dependent PL spectra peaked at about 521 nm or 2.38 eV. Obviously, the exciton with an energy of 2.38 eV participated in the PL process and had the largest radiative recombination rate. That means the 2.38 eV energy state contributed to the PL intensity the most. Then, we normalized Figure 2b at the vertices to obtain the inset in Figure 3a. Through careful observation, we found that the difference among the normalized PL spectra showed two peaks at 2.24 and 2.48 eV, where the relative PL intensity changed significantly with temperature. This result indicates that two excitons with energies of 2.24 and 2.48 eV were also involved in the PL process. The contributions of the three excitons to PL intensity changed with the varying temperature. Additionally, we obtained the normalized PL spectra which originated from the recombination of the involved three excitons with different energy states, as shown in Figure 3a. Namely, there were only three types of excitons with the energies of 2.24, 2.38, and 2.48 eV, which participated in the PL process in the [MnBr_4_]BrCs_3_ crystal.

The three spectra were used to fit the PL spectra of [MnBr_4_]BrCs_3_ at 78 K and 500 K. As shown in Figure 3b, it can be considered that the PL spectra at different temperatures were linearly combined by the luminescence spectra of these three energy states.

In addition, unlike other lead-based perovskite quantum dots [22], lead-free single crystals [23], and organic–inorganic halide clusters [24], the emission wavelength of $\lambda_{em}$ had no obvious red shift or blue shift (△$\lambda_{em}$ ≤ 2 nm) in the whole measurement temperature range (78 K~500 K). It was revealed that the green luminescence peak position of [MnBr_4_]BrCs_3_ single crystal was independent of temperature, similar to the phosphorescence of many heavy metal complexes [25]. Figure 2d shows the dependence of PL integral strength on temperature, in which the red dotted line corresponds to Figure 2b and the black dotted line corresponds to Figure 2c, indicating that the heating and cooling process did not affect these characteristics and that this material had excellent temperature stability. To explain these phenomena, the experimental results are explained according to the Boltzmann distribution theory [26], in which the solid line in Figure 2e shows the fitting results, and the results show the models of excitons with three different energies participating in the luminescence process, which are used to fit the experimental results of photoluminescence spectral intensity and peak position varying with temperature.

**Figure 4:**
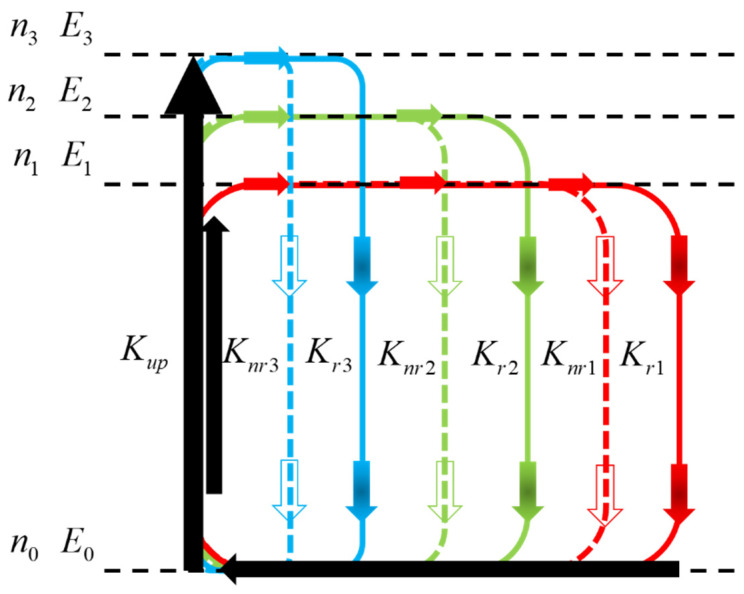
The model of temperature-dependent PL emission. See the text for the meaning of the symbols.

As shown in Figure 4, in the metal halide anti-perovskite crystal material [MnBr_4_]BrCs_3_, the unexcited [MnBr_4_]BrCs_3_ is in the ground state, marked as $E_{0}$. If [MnBr_4_]BrCs_3_ is excited, the excited electrons are still related to the remaining holes and would be either in the same lattice site or in two adjacent sites. When [MnBr_4_]BrCs_3_ is excited, there are a large number of energy levels in the solid film. Normally, according to the interval between adjacent energy levels, the energy levels can be sorted into i types. These energy levels can be labeled as $E_{1},\;E_{2},\ldots ,\;E_{j},\;\ldots ,\;E_{i}$. Assuming that the total number of lattice sites of a given material is n, the number of these sites in the $E_{j}$ state is $n_{j}$, then:

(1) $$n=\sum_{j=1}^{i}n_{j}$$

where $n_{0}$ is the number of sites (or molecules) in the ground state $E_{0}$. The energy difference between a given energy level and $E_{1}$ is defined as $E_{1j}=E_{j}-E_{1}$, where j=1,2,3,…,i. To simplify the model, the total pumping rate from the ground state to all excited states is denoted as $K_{up}$, which is assumed to be a constant independent of temperature. The radiative and non-radiative recombination rates of excitons in the $E_{j}$ state are denoted as $K_{rj}$ and $K_{nrj}$, respectively (j=1,2,3,…,i). At the same time, the pumping and recombination maintain balance dynamically during all PL measurements. From Equation (1), the pumping and recombination are balanced as follows:

(2) $$n_{0}K_{up}=\sum_{j=1}^{i}n_{j}(K_{rj}+K_{nrj})\;\;(j=2,3,\ldots ,i)$$

Obviously, the excitons of different states obey the Boltzmann distribution.

(3) $$n_{j}=n_{1}e^{-E_{1j}/K_{B}T}$$

where $K_{B}$ is Boltzmann’s constant, T is the absolute temperature measured on the sample, and the PL intensity should be:

(4) $$I=\sum_{j=1}^{i}n_{j}K_{rj}\;\;(j=1,\;2,\;\ldots ,\;i)$$

From Equations (1)–(4), we can get:


(5) $$I=I_{0}\frac{1+A_{2}e^{-E_{12}/K_{B}T}+\cdots +A_{j}e^{-E_{1j}/K_{B}T}}{1+C_{2}e^{-E_{12}/K_{B}T}+\cdots +C_{j}e^{-E_{1j}/K_{B}T}}\overset{}{}\;\;(j\;=\;2,\;3,\;\ldots ,\;i)$$

$I_{0}=\frac{nK_{up}K_{r1}}{K_{up}+K_{r1}+K_{nr1}}$, which is proportional to the total number of molecules n, is a coefficient related to the pumping rate and total recombination rate of excitons in the lowest excited state $E_{1}$; $A_{j}$ is the ratio of the radiative recombination rate of the $E_{j}$ state exciton to the radiative recombination rate of the $E_{1}$ state exciton, defined as $A_{j}=\frac{K_{rj}}{K_{r1}}\;\left(j\;=\;1,\;2,\;\ldots ,\;i\right)$. A larger $A_{j}$ means that the $E_{j}$ exciton has a greater radiative recombination rate than the $E_{1}$ exciton. $C_{j}=\frac{K_{up}+K_{rj}+K_{nrj}}{K_{up}+K_{r1}+K_{nr1}}\;\left(j=1,\;2,\;\ldots ,\;i\right)$ is a constant, which is related to the pumping rate and total recombination rate of $E_{j}$ and $E_{1}$ excitons. For example, according to this experiment, the energy levels of [MnBr_4_]BrCs_3_ could be sorted into three groups, namely i=3; then, Equation (5) is:

(6) $$I=I_{0}\frac{1+A_{2}e^{-E_{12}/K_{B}T}+A_{3}e^{-E_{13}/K_{B}T}}{1+C_{2}e^{-E_{12}/K_{B}T}+C_{3}e^{-E_{13}/K_{B}T}}$$

From Equation (3):

(7) $$n_{1}:n_{2}:n_{3}=1:e^{-\frac{E12}{K_{B}T}}:e^{-\frac{E13}{K_{B}T}}$$

and:

(8) $$I_{1}:I_{2}:I_{3}=1:A_{2}e^{-\frac{E_{12}}{K_{B}T}}:A_{3}e^{-\frac{E_{13}}{K_{B}T}}$$

where $I_{j}\;\left(1\leq j\leq i\right)$ is the intensity of PL produced by exciton recombination in the state $E_{j}$.

The experimental data were fitted by Equation (6). In Figure 2e, the solid line is the fitting result of the PL integrated intensity of [MnBr_4_]BrCs_3_ under varying temperatures. The fitting parameters are shown in Table 1.

It can be seen from Figure 3 that [MnBr_4_]BrCs_3_ has three energy states with energies that peaked at 2.24 eV, 2.38 eV, and 2.48 eV. Its energy difference, $E_{12}$ and $E_{13}$, exactly corresponded to the energy of $E_{12}$ (143.92 meV) and $E_{13}$ (241.48 meV) in Table 1. Therefore, it can be considered that the PL spectra at different temperatures were formed by the linear combination of the PL spectra of the three energy states, namely:

(9) $$I=w_{1}I_{1}+w_{2}I_{2}+w_{3}I_{3}$$

where $w_{1}$, $w_{2}$, and $w_{3}$ are the coefficients of the three resolved spectra. 

**Figure 5:**
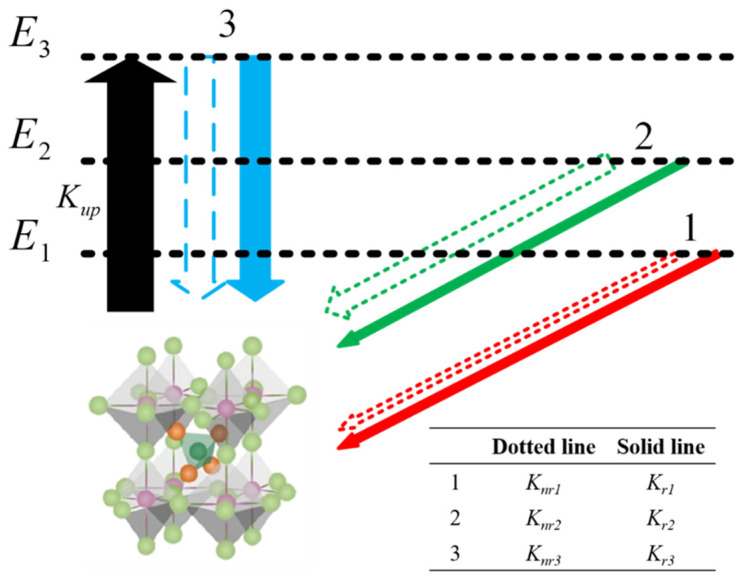
Schematic illustration of charge separation excitons in different states.

The best fitting result was i=3. It can be seen from the inset of Figure 3a that two luminescence peaks varied greatly with temperature on the left and right of the peak at 2.38 eV, indicating that there were three states of PL of the [MnBr_4_]BrCs_3_ film. Figure 5 shows the generation and recombination diagram of excitons in different energy states of [MnBr_4_]BrCs_3_. Assuming that $K_{up}$ remains unchanged, as shown by the green arrow line in Figure 5, the exciton radiative recombination rate and non-radiative recombination rate in $E_{1}$ state were recorded as $K_{r1}=1$ and $K_{nr1}=1$. It can be seen from $A_{2}=\frac{K_{r2}}{K_{r1}}=1$ and $C_{2}=\frac{K_{up}+K_{r2}+K_{nr2}}{K_{up}+K_{r1}+K_{nr1}}=38$ that $K_{r2}=K_{r1}$ and $K_{nr2}$ were almost 38 times the value of $K_{nr1}$, indicating that the radiative recombination rate of the $E_{2}$ exciton state was approximately equal to the $E_{1}$ exciton state, while the non-radiative recombination rate was very high, about 38 times that of $E_{1}$ exciton state, and the energy of the $E_{2}$ state was 143.92 meV higher than that of the $E_{1}$ state. $K_{r3}$ was about 64 times that of $K_{r1}$, and $K_{nr3}$ was about 66 times that of $K_{nr1}$. The values $A_{3}=64$ and $C_{3}=66$ indicate that the radiative recombination rate of the $E_{3}$ exciton state was 64 times that of the $E_{1}$ exciton state, while the non-radiative recombination rate was about 66 times greater. The energy of the $E_{3}$ state was 241.48 meV higher than that of the $E_{1}$ state.

**Figure 6:**
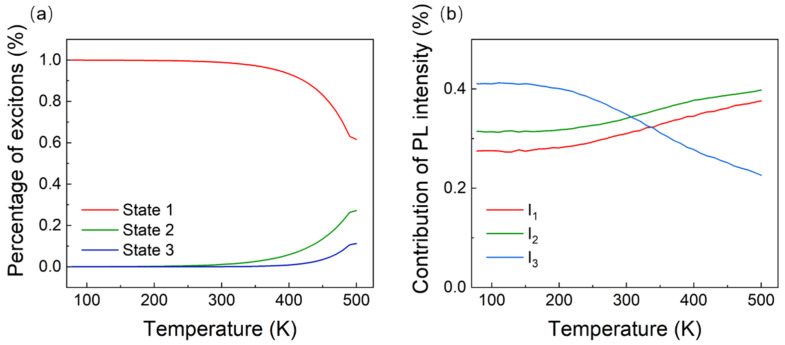
(**a**) Variation in the percentages of the three types of excitons in a [MnBr_4_]BrCs_3_ film with increasing temperature. (**b**) Variation of the ratio of the PL intensity contributed from the three types of excitons in a [MnBr_4_]BrCs_3_ film with increasing temperature.

To obtain more information, the percentage of each exciton state in the PL was calculated according to Equation (7) and the above fitting parameters. As shown in Figure 6a, more than 99% of the excitons were located in the $E_{1}$ state from 78 to 300 K. With the increase in temperature from 300 to 500 K, the exciton percentages of $E_{2}$ and $E_{3}$ states increased rapidly (for example, at 330 K, the exciton percentages of $E_{1}$, $E_{2}$, and $E_{3}$ states were 98%, 1.8%, and 1.2%, respectively). At 430 K, they were 88.3%, 9.7%, and 2%, respectively. The percentage of excitons in the $E_{2}$ state increased by almost 5 times). In the range of 78 to 250 K, most of the light emission came from the recombination of the $E_{1}$ state excitons, which caused the PL intensity to change slightly with the temperature. With increasing temperature, the percentage of the $E_{1}$ exciton decreased slightly from 300 to 500 K. Although the radiative recombination rate of the $E_{3}$ state was about 64 times that of the $E_{1}$ state, the non-radiative rate was about 66 times greater, so it did not lead to an increase in PL intensity. As the temperature increased from 400 to 500 K, the percentage of $E_{1}$-state excitons decreased rapidly, while the proportion of $E_{2}$- and $E_{3}$-state excitons increased rapidly. This trend slowed down as the temperature neared 500 K, which may have been due to the increase in non-radiative transition caused by lattice vibration and lattice relaxation of luminescence center due to the temperature being too high [27].

Figure 6b shows the contribution percentages of $I_{1}$, $I_{2}$, and $I_{3}$ (i.e., the PL intensities generated by the excitons in $E_{1}$, $E_{2}$, and $E_{3}$ states, respectively). The red solid line corresponds to $I_{1}$, the green solid line corresponds to $I_{2}$, and the blue solid line corresponds to $I_{3}$. At 78 K, $I_{3}$ had the highest contribution ratio of 41%, while the ratios for $I_{1}$ and $I_{2}$ were 28% and 31%, respectively. With a gradual rise in temperature, at 310 K, the contribution rate of $I_{3}$ was equal to the contribution rate of $I_{2}$ (34%); at 340 K, the contribution rate of $I_{3}$ dropped to 32%, which is equal to the contribution rate of $I_{1}$. When the temperature was increased to 500 K, the contribution rate of $I_{3}$ dropped to 23%, and the contribution rates of $I_{1}$ and $I_{2}$ increased to 38% and 39%, respectively. This may be because the excitons could not be disconnected by the weaker thermal vibration at a lower temperature; then, the excitons tended to form the luminescent centers. As the temperature increases, the exciton spectrum of the high-energy state will broaden the PL peak, while the PL intensity will decrease. The excitons can more easily participate in heating or conduction. Therefore, in many semiconductor materials, clear exciton emission can only be observed at low temperatures, and when the temperature rises, the excitons decompose due to thermal excitation and may even be quenched [28,29]. However, because this paper involves a complex structure crystal composed of three elements, there are at least two more light-emitting states. Even excluding the interaction of the three light-emitting states, at least three such equations are needed to explain the change of the light-emitting intensity of the three light-emitting states with temperature. Because the structure of [MnBr_4_]BrCs_3_ material is tetrahedral and separated by a three-dimensional octahedral framework, the exciton may be excited within the tetrahedral structure, the octahedral framework, or between them. Further deep investigations are needed if the three excitons have to be attributed to three transitions exactly. Similar phenomena were found by Karunadasa H. I. et al. [30]. They found that many luminescence states coexisted in (N-MEDA) [PbBr_4_] thin films when the film was excited by terahertz technology. Through the equation deduced in the present work, the involved types of excitons, together with their energy states and radiative- or non-radiative-recombination rates, can be found only by the simulation of temperature-dependent PL spectra, and some details in the PL process need not be considered. 

Although the above analysis is aimed at [MnBr_4_]BrCs_3_, we believe that Equation (5) is also applicable to other exciton-emitting materials. For example, when i=2, $A_{2}=0$, then Equation (5) becomes:

(10) $$I=\frac{I_{0}}{1+C_{2}e^{-E_{12}/K_{B}T}}$$

This is the exact expression of the temperature-dependent luminous intensity of inorganic semiconductors with exciton emission [31], which also proves the validity of the current model from another perspective.

## 4. Conclusions

We studied the temperature-dependent photoluminescence of a [MnBr_4_]BrCs_3_ film, and the results showed that the material had excellent stability and repeatability at different temperatures. According to the principle of photoluminescence, the luminescence intensity and luminescence peak were fitted, and the temperature-dependent PL spectra were resolved to obtain the luminescence spectra of excitons at three energy states. The calculation showed that the spectra measured at the temperature ranging from 78 to 500 K could be obtained by the linear combination of the luminescence spectra of the three types of excitons. The experiment also showed that there were three types of excitons at different energy levels involved. It was found that the radiative recombination rate of exciton at the $E_{2}$ state was about equal to that of the $E_{1}$ state, while the non-radiative recombination rate was very high—about 38 times that of $E_{1}$ state. At the same time, the radiative recombination rate of the $E_{3}$ state was about 64 times that of the $E_{1}$ state, while the non-radiative recombination rate was about 66 times that of the $E_{1}$ state, meaning that the photoluminescence efficiency was very high. Excluding the influence of water, the [MnBr_4_]BrCs_3_ film could be used as a good photoluminescence material. Because almost 99% of excitons were in the $E_{1}$ state at room temperature, which had high photoluminescence efficiency, few electrons and holes could be formed and connected to form excitons to participate in electroluminescence. This may have been the reason for the difficulty in the realization of electroluminescence devices with [MnBr_4_]BrCs_3_ films.

## Figures and Tables

**Table 1:** Fitting parameters of the PL spectra of [MnBr_4_]BrCs_3_ film.

Material	Exciton Labels	$A_{j}=\frac{K_{rj}}{K_{r1}}$	$C_{j}=\frac{K_{up}+K_{rj}+K_{nrj}}{K_{up}+K_{r1}+K_{nr1}}$	$E_{1j}\;(\mathrm{meV})$
[MnBr_4_]BrCs_3_	1	1	1	0
	2	1	38	143.92
	3	64	66	241.48

## Data Availability

Not applicable.
